# Lifestyle Variables Do Not Predict Subjective Memory Performance Over and Above Depression and Anxiety

**DOI:** 10.3389/fpsyg.2020.00484

**Published:** 2020-03-19

**Authors:** Anna Mascherek, Nathalie Werkle, Anja S. Göritz, Simone Kühn, Steffen Moritz

**Affiliations:** ^1^Department of Psychiatry and Psychotherapy, University Medical Center Hamburg-Eppendorf (UKE), Hamburg, Germany; ^2^Department of Psychology, Albert-Ludwigs-Universität Freiburg, Freiburg im Breisgau, Germany; ^3^Lise Meitner Group for Environmental Neuroscience, Max Planck Institute for Human Development, Berlin, Germany

**Keywords:** metacognition, lifestyle variables, middle adulthood, attention, memory

## Abstract

The diagnostic value of subjective cognitive complaints for cognitive functioning in a clinical setting remains unresolved today. However, consensus exists on the relation between subjective cognitive complaints (SCC) and mood variables such as anxiety and depression. Hence, SCC have also been discussed as potential proxies of psychopathology rather than representing cognitive functioning. In order to shed more light on yet still unexplained variance in subjective cognitive complaints, the relation between lifestyle variables (such as nutrition habits, exercise, alcohol consumption, smoking, quality of sleep, and Body Mass Index) and subjective complaints of selective attention as well as subjective memory performance were assessed, additionally to the influence of objective memory performance, measures of anxiety, and depression. A sample of 877 (554 women) healthy, middle-aged individuals (51 years on average, age range 35–65) was assessed in the present study. In a logistic regression framework results revealed that the effect of lifestyle variables on subjective complaints of selective attention as well as subjective memory performance was rendered non-significant. Instead, subjective complaints of selective attention and subjective memory performance were significantly determined by measures of both, anxiety and depression. One unit increase in anxiety or depression led to an increase of 6 or 15% in subjective memory performance complaints, respectively. For subjective complaints of selective attention, a one unit increase in anxiety or depression led to an increase of 11 or 26%, respectively. The strong relation between SCC and measures of depression and anxiety corroborates the notion of SCC being indicative of mental health and general well-being.

## Introduction

The diagnostic value of subjective cognitive complaints (SCC) for the evaluation of cognitive functioning remains unsatisfactory to date. Although SCC in combination with objective memory impairment are a necessary criterion for the diagnosis of Mild Cognitive Impairment (MCI), subjective and objective impairment do not necessarily match (Moritz et al., 2004).

### Predictive Value of Subjective Cognitive Complaints – Early Warning Sign or Epiphenomenon?

Existing studies report brain atrophy resembling Alzheimer dementia (AD)-disease in mood-disorder-free, older individuals with SCC but (yet) without objective cognitive deficits (Saykin et al., 2006; Chao et al., 2010; Jessen et al., 2010). These studies support the notion of SCC being of diagnostic value for (early) cognitive deficits (Jessen et al., 2014). The significance of SCC for objective memory performance, however, remains subject of controversy. Amariglio et al. (2011) showed that cognitive performance could be related specifically to SCC, depending on the way SCC were assessed. The authors assessed SCC targeting different domains (e.g. getting lost in familiar streets, change in ability to remember things, trouble following a conversation) and related them cross-sectionally to objective cognitive measures. While they found that on the one hand the number of SCC was negatively related to task performance, they also found that the relation between SCC-items and cognitive performance varied in strength depending on SCC-item. While SCC in older individuals could be a stronger indicator for cognitive impairment, they could more likely mirror mood-related states in younger individuals. From a review of studies, Jonker et al. (2000) conclude that only in the “oldest old,” SCC on every day events should be taken as a valid early sign of cognitive decline. In contrast, even in the “younger-old”, SCC more likely reflect mood and personality. Adding to that, Derouesné et al. (1999) analyzed SCC in older as well as younger adults. Here, correlates of SCC resembled each other across age (two samples aged 39 and 61 years on average, respectively), with the strongest relation to anxiety-related symptoms in both groups. The authors conclude that “memory complaints of elderly do not appear basically different from memory complaints of younger subjects. They constitute a complex psychological symptom unlikely to be explained by […] memory performance” (Derouesné et al., 1999, abstract, last sentence, p. 291). A meta-analysis by Mitchell et al. (2014) came to the conciliatory conclusion that SCC do not necessarily need to be related to objective memory performance at the time of assessment; however, they are justifiably in a diagnostic process as the conversion rate to dementia or MCI is twice as high in older individuals with SCC prior to onset.

### Subjective Cognitive Complaints and Lifestyle Variables

Despite the ongoing debate of the possible relation between SCC and objective memory performance, it is generally agreed upon that SCC are related to mood disorders (Jonker et al., 2000; Jorm et al., 2004; Elfgren et al., 2010; Buckley et al., 2013; Mitchell et al., 2014). While SCC have consistently been associated with mood variables, these variables do still not explain all the variance in SCC. If SCC can generally be attributed to self-rated health and well-being (Jorm et al., 2004; Mewton et al., 2014), the analyses of additional non-cognitive variables that are known to influence well-being and health emerges as an obvious question. This question has indeed sparked the interest of some research groups. Mewton et al. (2014) examined a population-based sample aged 65–85 years with respect to a multitude of lifestyle variables and SCC. About one third of the participants reported SCC, which was related to general distress, low functioning, and negative mental and physical health, rather than objective memory performance. In a similar vein, Paradise et al. (2011) studied lifestyle variables and SCC in an Australian sample of middle-aged adults (45–65 years). Here, SCC were again rather related to psychological distress than indicating cognitive (mal)performance and warranted further examination of general well-being and health.

Quality of sleep and stress level have also been analyzed in individuals with SCC. Poor sleep quality and preoccupation with business were related to SCC, however, not to objective memory performance in a study by Miley-Akerstedt et al. (2018). This was also shown by Kang et al. (2017), where poor quality of sleep was related to SCC in healthy older individuals. Instead, Saint Martin et al. (2012), found a relationship between SCC with sleep medication intake as well as anxiety and depression but not with quality of sleep.

Nutrition habits and its relation to SCC have been under study as another lifestyle variable. In a large longitudinal study, Bhushan et al. (2018) found adherence to Mediterranean diet representing a protective factor against subjective cognitive decline. Generally, long term adherence to Mediterranean diet seems to offer protective aspects, which includes the consumption of fish, fruits and vegetables, unsaturated fat, and little meat. Fish has also been specifically addressed and found to differentially exert a protective influence (Boespflug et al., 2016).

Turning to yet another lifestyle variable, Lee et al. (2013) analyzed the relation between physical activity in household, work, and leisure and SCC in middle-aged Americans. They found moderate household-related physical activity being associated with increased SCC, while moderate levels of leisure-time related physical activity were associated with less SCC. The authors explain these opposing results as household being perceived as duty or even burden and might, hence, promote depressive thoughts and feelings, which, in turn has been shown to exert a detrimental effect on SCC. Leisure-time related physical activity, however, might rather reflect fun and recreation, and, hence, be related to psychological well-being.

Against the background of the existing literature one might conclude that the presence of SCC indicates the need of further clinical assessment rather than representing cognitive functioning. Because of their relation to anxiety disorders, psychological distress, service use for mental health problems and, partly, even to alcohol disorders, SCC could be proxies of psychopathology rather than pure cognitive functioning.

Aim of the present study was to tackle the question of whether lifestyle variables represent suitable predictors of SCC in a large, middle-aged, population-based, German sample in order to add to the understanding of what SCC truly reflect. We explicitly aimed at a middle-aged sample. Studies have examined the relation between SCC and lifestyle-variables in middle-age, however, not with a comparable plethora of variables and not with a German representative sample (Paradise et al., 2011; Lee et al., 2013). In order to better understand what SCC reflect, we consider it important to also focus on middle age, especially because studies exist that show that lifestyle in middle adulthood does effect cognitive aging in old age.

We analyzed whether the lifestyle factors quality of sleep, eating habits, physical activity, Body Mass Index (BMI), alcohol consumption, craving for alcohol, and smoking were related to SCC. Also, depression and anxiety-measures were included into the analyses. We expected to find a substantial effect of depression and anxiety-measures. Additionally, we expected to find a protective influence of sport, healthy diet and healthy sleep, as well as a negative influence of smoking, alcohol consumption, craving, and higher BMI.

## Materials and Methods

The sample comprised 877 middle-aged individuals (mean age 50.8 years; SD 8.5 years; range 35–65 years; 554 (63%) were female) from an online assessment conducted between March 21 and March 31, 2015. Participants were recruited via WiSoPanel, an online-platform for collecting data from a general population-based sample. WiSoPanel is a tool to conduct high powered, anonymous studies with participants drawn from diverse sources in order to reduce selection bias. The platform comprises participants with different socio-economic background and resembles the general population in typical demographic characteristics (Göritz, 2009, 2014). Participation was rewarded with a manual on relaxation exercises upon completion of the study.

### Measures

#### Subjective Cognitive Complaints

Subjective cognitive complaints were assessed via two self-rated questions on a four-point Likert scale ranging from “not at all,” “a little,” “often true,” and “absolutely true.” The two questions addressed self-rated memory performance and self-rated selective attention. Exact wording, answering categories, and descriptive statistics are displayed in Table 1. Individuals rating their memory or selective attention with “not at all” or “a little” were classified as not reporting SCC, individuals rating “often true” or “absolutely true” were classified as individuals with complaints. This classification has been applied previously (Paradise et al., 2011). Single-item assessment was preferred over the use of an established questionnaire in the present study due to feasibility.

**TABLE 1:** Self-reported subjective cognitive complaints (*N* = 877).

	**Absolute frequency**	**Percent**
**I am bad at memorizing new contents**		
Not at all	329	37.5
A little	429	49
Often true	91	10.4
Absolutely true	28	3.1
**I have difficulties concentrating**		
Not at all	412	47
A little	351	40
Often true	77	8.8
Absolutely true	37	4.2

#### Objective Memory Performance

Memory was assessed with the online-adaptation of the German adaptation of the Auditory Verbal Learning Test (AVLT/VLMT; Helmstaedter and Durwen, 1990). A wordlist with 15 words was presented two times in a row one word at a time on the screen. After both learning trials, participants had to type the words they remembered in an input field on the computer display in any order. After participants answered a questionnaire on lifestyle variables developed by two members of the research team (SM, NW), a third delayed recall of the wordlist was requested. Delayed recall was used to assess objective memory performance in the present study.

### Mood

Depression was assessed as sum score of nine items from the German version of the Patient Health Questionnaire (PHQ; Gräfe et al., 2004). The items of the 4-point Likert-scale (not at all, rarely, more than half of the days, almost every day) assess the presence of DSM-IV-criteria for major depression referring to aspects within the last 2 weeks. Internal consistency of the German version is good with Cronbach’s α = 0.88 (Gräfe et al., 2004).

Anxiety was assessed as a sum score of five items from the German Version of the Penn State Worry Questionnaire (PSW; Glöckner-Rist and Rist, 2014). Items from the PSW were “Many situations make me worry,” “I know I should not worry about things, but I just cannot help it,” “When I am under pressure, I worry a lot,” “I have been a worrier all my life,” and “I notice that I have been worrying about things.” Items were to be answered on a 5-Point Likert-scale ranging from “not at all typical of me” to “very typical of me.”

### Lifestyle Variables

Physical activity was assessed as dichotomous variable, representing sport/no sport.

Alcohol consumption was assessed using the following three items of the Alcohol Use Disorder Identification Test Consumption (AUDIT-C; Bush et al., 1998): Frequency of drinking (ranging from never to four times or more per week), number of consumed beverages (none to up to ten or more drinks per day), as well as how often more than six drinks are consumed on any drinking occasion (never to daily). As an additional measure describing alcohol consumption, we asked for craving (“How strong was your desire to consume alcohol within the last 7 days” ranging from “not existent” to “very strong”). Smoking was assessed as number of cigarettes smoked per day.

Frequency of meat, fish, and fruit and vegetable consumption were considered for the concept of “Mediterranean diet” (daily, 3–5 times/week, 1–2 times/week, rarely/never). Moreover, we assessed the attention paid to fat intake and the attention paid to carbohydrate intake on a 4-Point Likert-scale (always, frequently, rarely, never). The Body Mass Index (BMI) as a measure of obesity as well as underweight and normal body weight was calculated as a composite score from height and weight (BMI = person’s weight in kilograms divided by his or her height in meters squared).

Sleep was assessed with two questions addressing falling asleep (“Do you have any problems falling asleep?”) and sleeping through (“Do you have any problems sleeping through the night?”) on a 4-Point Likert-scale ranging from “never” to “almost every day.”

### Analyses

We analyzed data in a hierarchical logistic regression framework. The hierarchical approach was chosen as we were interested in the potential additional exploratory value of lifestyle variables over and above the common measures objective performance, demographic variables, and mood. As predictors for our two subjective complaints variables, memory performance, sex, age, and education as control variables were included into the model in a first step. Educational level was assessed via years of formal education. We then added depression and anxiety. Smoking, alcohol consumption, craving, physical activity, BMI, amount of meat, fruits and vegetables, fish and attention paid to consumption of carbohydrates and fat, as well as quality of sleep as lifestyle variables were subsequently added to the model. All analyses were conducted using SPSS (Version 26).

## Results

A total of 13.5% our sample reported memory-related SCC. For attention-related SCC, this was true for 13% of the sample (see Table 1). Means and standard deviations (SD) for lifestyle and control variables are depicted in Table 2. A correlational table of the predictor variables can be seen in Table 3.

**TABLE 2:** Descriptive statistics [means and standard deviation (SD)] of the study variables.

**Variable**	**Mean**	**SD**	**Percent**
Objective memory performance*	10.6	3.3	
Sex			63.2 women
Age (in years)	50.8	8.5	
Education	13.6	3.8	
Depression (phq)	13.6	4.6	
Anxiety (PSW)	12.2	5.4	
Smoking (cigarettes per day)	4.1	8.1	
Alcohol consumption	6.4	1.9	
Physical activity (sport at all or no sport)			51.3 yes
craving	0.5	0.9	
**Nutrition**			
Fat intake**	2.4	1.0	
Carbohydrate intake**	2.1	1.0	
Fruits and vegetables***	3.4	0.8	
Meat***	2.2	0.8	
Fish***	1.7	0.627	
BMI	27.3	6.4	
Problems sleeping through	2.1	0.9	
Problems falling asleep	1.8	0.8	

* = objective memory performance was assessed as delayed word-list recall from the Auditory Verbal Learning Test, means and standard deviation represent remembered words; ** = Attention paid to intake; *** = Consumption of; PHQ-9 = patient health questionnaire 9 (depression); PSW = Five items from the German Version of the Penn State Worry Questionnaire; BMI = Body Mass Index.

**TABLE 3:** Correlational table of predictor variables.

	**1**	**2**	**3**	**4**	**5**	**6**	**7**	**8**	**9**	**10**	**11**	**12**	**13**	**14**	**15**
1.Memory	−														
2.Age	−0.13*														
3.Education	0.21*	–0.03													
4.Depression	−0.11*	−0.12*	−0.11*												
5.Anxiety	−0.07*	−0.14*	−0.10*	0.65*											
6.Smoking	–0.01	0.07*	−0.19*	0.12*	0.08*										
7.Alcohol	–0.04	0.03	–0.001	0.02	–0.03	0.10*									
8.Craving	−0.14*	–0.04	0.03	0.17*	0.12*	–0.03	0.26*								
9.Fat	0.03	0.12*	0.07*	–0.01	0.03	−0.17*	−0.09*	0.03							
10.Carbohydrate	–0.04	0.1*	0.03	–0.02	0.01	−0.11*	–0.06	–0.02	0.66*						
11.Fruits and vegetable	0.17*	0.05	0.20*	−0.13*	−0.09*	−13*	−0.08*	−0.07*	0.25*	0.20*					
12.Meat	−0.12*	–0.02	0.01	–0.06	−0.09*	0.07*	–05	0.04	−0.16*	−0.11*	–0.03				
13.Fish	–0.05	0.11*	–04	−0.10*	–0.07	–0.05	–0.02	0.05	0.13*	0.15*	0.15*	0.06			
14.BMI	−0.07*	0.13*	−0.12*	−0.08*	0.07*	−0.10*	0.05	–0.04	0.08*	0.11*	−0.07*	0.19*	0.02		
15.Sleeping 1	0.02	0.08*	–0.04	0.46*	0.30*	0.04	–0.02	0.04	0.04	–0.02	0.01	–0.01	–0.02	0.01	
16.Sleeping 2	0.08*	0.01	−0.14*	0.42*	0.35*	0.13*	0.05	0.07	0.02	–0.02	−0.07*	–0.02	–0.03	0.05	0.52*

* = *p* < 0.05; memory = objective memory performance assessed as delayed word-list recall from the Auditory Verbal Learning Test; Depression = assessed with the PHQ-9 (patient health questionnaire 9); Anxiety = assessed as five items from the German Version of the Penn State Worry Questionnaire (PSW); Smoking = cigarettes per day; alcohol = Alcohol consumption according to Alcohol Use Disorder Identification Test; Craving = Desire to consume alcohol; Fat and Carbohydrate = Attention paid to intake each; Fruits and Vegetables, Meat, and Fish = Consumption of each; BMI = Body Mass Index; Sleeping 1: Problems sleeping through; Sleeping 2: Problems falling asleep.

We started with a first model, entering objective memory performance, sex, age, and education into our first model (Model I, Table 4). The overall model for self-reported memory complaints and self-reported complaints for selective attention yielded a significant result (*χ*^2^ = 12.706, *df* = 4, *p* < 0.05, *n* = 877 and (*χ*^2^ = 32.338, *df* = 4, *p* < 0.05, *n* = 877, respectively). Concerning the individual predictors, memory performance had a significant effect [memory complaints: odds ratio of 0.93 (95% CI = 0.87–0.99), complaints selective attention: odds ratio of 0.91 (95% CI = 0.85–0.97)], indicating that better objective memory predicted less SCC. However, as odds ratios are very close to 1, the effect remains small. Male sex had a significant effect on memory complaints only [memory complaints: odds ratio of 1.74 (95% CI = 1.12–2.72), complaints selective attention: odds ratio of 1.38 (95% CI = 0.88–2.18)] indicating that males had a significant tendency to report more memory-related SCC. Age and education were no significant predictors for self-reported memory complaints, however, did significantly predict self-reported complaints in selective attention [Age: odds ratio of 0.97 (95% CI = 0.95–0.99); Education: odds ratio of 0.93 (95% CI = 0.85–0.96)]. Nagelkerke’s *R*-squared for self-reported memory complaints was 0.03 which, according to Cohen, reflects a small effect. For self-reported complaints in selective attention, Nagelkerke’s *R*-squared was 0.07, which reflects a medium effect.

**TABLE 4:** Results of regression analyses.

**Predictor**	**Model**					
	**I**		**II**		**III**	
	**Odds Ratio (95% CI)**		**Odds Ratio (95% CI)**		**Odds Ratio (95% CI)**	
	**mem**	**con**	**mem**	**con**	**mem**	**con**
Memory	**0.93 (0.87–0.99)**	**0.91 (0.85–0.97)**	0.96 (0.90–1.03)	0.95 (0.88–1.03)	0.96 (0.89–1.03)	0.94 (0.86–1.02)
Sex (male)	**1.74 (1.12–2.72)**	1.38 (0.88–2.18)	1.39 (0.86–2.26)	0.92 (0.53–1.6)	1.51 (0.88–2.6)	0.86 (0.46–1.6)
Age	0.99 (0.96–1.01)	**0.97 (0.95–0.99)**	1 (0.98–1.03)	0.99 (0.96–1.02)	1.01 (0.99–1.04)	0.99 (0.96–1.03)
Education	0.99 (0.93–1.04)	**0.90 (0.85–0.96)**	1 80.95–1.07)	**0.92 (0.86–0.99)**	1.01 (0.95–1.08)	**0.92 (0.85–1)**
Depression			**1.15 (1.09–1.21)**	**1.26 (1.19–1.34)**	**1.17 (1.1–1.24)**	**1.28 (1.2–1.37)**
Anxiety			**1.06 (1.01–1.11)**	**1.11 (1.05–1.18)**	**1.06 (1.01–1.12)**	**1.12 (1.06–1.19)**
Smoking					0.98 (0.95–1.01)	1.03 (0.996–1.06)
Alcohol consumption					0.93 (0.83–1.05)	0.89 (0.78–1.02)
Physical activity (yes)					1.07 (0.67–1.71)	0.81 (0.46–1.4)
Craving					1.02 (0.80–1.3)	1.02 (0.78–1.33)
Carbohydrate					0.81 (0.60–1.10)	0.89 (0.62–1.27)
Fat					0.90 (0.67–1.21)	0.95 (0.66–1.37)
Meat					1.10 (0.82–1.50)	1.15 (0.82–1.62
Fish					0.69 (0.47–1.02)	0.65 (0.41–1.01)
Fruit and vegetables					0.95 (0.71–1.26)	1.23 (0.88–1.73)
BMI					0.98 (0.95–1.02)	0.99 (0.95–1.03
Problems falling asleep					1 (0.74–1.35)	0.83 (0.6–1.16)
Problems sleeping through					0.90 (0.68–1.12)	1.01 (0.74–1.38)

*Numbers in bold represent *p* < 0.05, 95% CI = 95% Confidence interval; mem = model parameter for subjective complaints on memory performance, con = model parameter for subjective complaints on selective attention. memory = objective memory performance assessed as delayed word-list recall from the Auditory Verbal Learning Test; Depression = assessed with the PHQ-9 (patient health questionnaire 9); Anxiety = assessed as five items from the German Version of the Penn State Worry Questionnaire (PSW); Smoking = cigarettes per day; alcohol = Alcohol consumption according to Alcohol Use Disorder Identification Test; Craving = Desire to consume alcohol; Fat and Carbohydrate = Attention paid to intake each; Fruits and Vegetables, Meat, and Fish = Consumption of each; BMI = Body Mass Index.*

The second model (Model II, Table 4) also included the mood-variables depression and anxiety. Compared to Model I, this led to a significantly better model fit for both, self-reported memory complaints and self-reported complaints in selective attention (*χ*^2^ = 92.743, *df* = 6, *p* < 0.05, *n* = 877; Δ*χ*^2^ = 80.038, Δ*df* = 4 for memory and *χ*^2^ = 216.753, *df* = 6, *p* < 0.05, *n* = 877, Δ*χ*^2^ = 184.414, Δ*df* = 4 for selective attention, respectively). Both, depression and anxiety led to more self-reported memory complaints [depression: odds ratio of 1.15 (95% CI = 1.09–1.21; anxiety: odds ratio of 1.06 (95% CI = 1.01–1.11)], which was also true for self-reported complaints in selective attention [depression: odds ratio of 1.26 (95% CI = 1.19–1.34; anxiety: odds ratio of 1.11 (95% CI = 1.05–1.78)]. Nagelkerke’s *R*-squared for self-reported memory complaints was 0.17 which, according to Cohen, reflects a strong effect. For self-reported complaints in selective attention, Nagelkerke’s *R*-squared was 0.41, which also reflects a strong effect. In adding mood-variables to the model, the effect for all demographic variables except for education in complaints in selective attention were reduced to non-significance. This indicates that the previously found effects were more precisely captured by depression and anxiety (see Table 4).

In a last model (Model III, Table 4), lifestyle variables were added (see “Materials and Methods” section for variables in detail). Neither for subjective memory performance nor subjective selective attention problems an effect of any lifestyle variable emerged (*χ*^2^ = 109.104, *df* = 18, *p* < 0.05, *n* = 877; Δ*χ*^2^ = 16.361, Δ*df* = 12 for memory and *χ*^2^ = 232.186, *df* = 18, *p* < 0.05, *n* = 877, Δ*χ*^2^ = 15.433, Δ*df* = 12 for selective attention, respectively). See Table 4 for details on single predictors.

## Discussion

In the present study we set out to test whether lifestyle variables were suitable predictors of SCC, explaining variance in subjective cognitive complaints concerning memory and selective attention in addition to measures of depression, anxiety, and objective memory performance. Generally, in our sample of middle-aged adults, SCC were present in about 13% of the individuals. This resembles numbers found in the literature (for example Paradise et al., 2011 on middle-aged adults).

### Demographics

In our study, only education was predictive of complaints about selective attention indicating that higher education serves as protective factor concerning subjective selective attention complaints. This is in line with results from Paradise et al. (2011), who analyzed SCC in a sample of middle-aged adults. In their study, male gender also exerted a negative effect on SCC, which could not be replicated in our study in the final model. The effect of educational differences accounting for cognitive complaints but neither age nor sex has been reported earlier (Mascherek et al., 2011). However, as that study comprised memory-clinic outpatients of old age (76 years of age on average), comparability to the present study must be questioned. Generally, demographic variables have been found to be predictive of SCC in some studies (Iliffe and Pealing, 2010; Paradise et al., 2011; Miley-Akerstedt et al., 2018), but not without controversy (Mewton et al., 2014). We speculate that demographic effects could be related differentially to SCC in different subdomains (such as memory or selective attention). Also, methodological reasons or design of the respective studies might explain those contradictory findings. This remains an open question that should be addressed in future research. It also remains unresolved whether this relation might differ in different age groups. The role of demographic variables is of special interest, as age and gender would almost act as predisposition due to their immutability, whereas education could be influenced. Potentially modifiable variables could then also be of interest from an intervention-perspective, especially if SCC reflect general (mental) health and well-being.

### Measures of Anxiety and Depression

In line with previous research, our measures for anxiety and depression explained a substantial amount of variance. In our sample, anxiety and depression scores differentially effected our variables. One unit increase in anxiety or depression led to an increase of 6 or 15% in subjective memory performance complaints, respectively. For subjective complaints of selective attention, a one unit increase in anxiety or depression led to an increase of 11 or 26%, respectively. Subjective selective attention was therefore influenced more strongly by mood variables than subjective memory performance. Previous research has already found differential effects on prospective and retrospective memory complaints (Mäntylä, 2003). Support for a differential effect also comes from a study by Amariglio et al. (2011), who report a varying relation between objective measures of memory performance and SCC, depending on how SCC were assessed. However, future studies should address this question of whether and how SCC are differentially influenced depending on cognitive domain addressed. One might speculate that attention as a more instable, elusive ability than memory performance, might be more strongly influenced by depression and anxiety. However, it is also possible that individuals tend to observe changes in selective attention more pronounced as this poses an impairment in ordinary day life that rather quickly becomes inconvenient.

### Lifestyle Variables

We were especially interested in whether lifestyle variables predict SCC in middle-aged adults above and beyond mood variables and objective memory performance. In our sample, none of the lifestyle factors proved influential. The variables for nutrition analyzed in our data were chosen to reflect Mediterranean diet. Mediterranean diet has been reported as protective in men (Bhushan et al., 2018). Also, especially fish consumption has been found to exert influence on SCC (Boespflug et al., 2016). With reaching marginal significance, consumption of fish was the variable closest of proving influential in our sample. Results do not warrant an in-depth discussion of a possible effect of fish consumption, however, point to the interesting road for future research to further analyze the effect of eating habits on SCC in middle-adulthood. Whether a real impact on SCC exists needs to be further evaluated in future studies.

Neither smoking, alcohol consumption, craving, physical activity, nor eating habits, BMI or quality of sleep had any relation to SCC. This has partly been documented differently in the literature, however, in a sample of older adults. In their study on lifestyle variables and SCC, Mewton et al. (2014) also did not find any effect of smoking, current drinking or physical activity. They do, however, report an influence of alcohol disorder, hence, alcohol consumption on a clinical level which was not assessed in the present study. They conclude that SCC rather reflect mental health and well-being than objective memory performance. Although our study focused on healthy individuals and middle adulthood, our interpretation follows that of previous research. Already in middle-aged, healthy adults, SCC are strongly related to mood-variables such as anxiety and depression. Hence, for clinical assessment, SCC might be valuable as a hint to take a closer look at mental health and well-being. We conclude that our results corroborate previous research that the diagnostic value of SCC is stronger for mental health than for cognitive impairment.

### Idiosyncratic Reference for the Evaluation of SCC

Due to the type of question by which SCC were assessed and the cross-sectional nature of the data in the present study, it cannot be decided whether those complaints reflect a worsening of individual cognitive performance over time or a general assessment of one’s own cognitive ability as a discrepancy to what is deemed normal or desirable. However, we argue that this uncertainty in our data does not reduce the impact of our work. It underlines the empirical finding that SCC are stronger related to mental condition and well-being than to memory performance. One might argue that SCC that are expressed without explicit social or self-reference rather reflect a self-evaluation that is anchored in one’s own general self-concept and not solely in memory performance. If the question was interpreted as self-evaluation, one might argue that the relation to objective memory performance should be higher, as a more or less realistic description of one’s own personal strengths and weaknesses in middle adulthood. If the question was interpreted as “complaint”, one would expect a stronger relation to mood variables as mood influences interpretation, especially with self-reference (Wisco, 2009; Everaert et al., 2017). As we examined a non-clinical sample, we would argue that a self-evaluation that also included the description of one’s own cognitive performance, would more strongly resemble objective measures than answering the question from the complaint point of view, as complaint might induce a mood-distorted view. This hypothesis, however, remains highly speculative and would need further evaluation. Also, the question of causality would need further evaluation and cannot, by any means, be answered from the data of the present study, as this would require longitudinal data. We do consider this question important, as this could aid understanding the existing controversy of SCC being related to objective measures or mood, respectively. Less so on a content-level but rather coming from a psychometric perspective, Rabin et al. (2015) already addressed the problem of lacking standards for the assessment of SCC and its possible implications on (non)comparability of study-results and inferences.

### Limitations

The results of our study are based on a large population-based sample, which represents a strength with respect to both, composition and size. A combination of lifestyle variables was assessed with the focus on general habits. While this aspect is a strength in the way that our data represent a realistic picture of lifestyle habits, it, on the downside, entails a low degree of precision for the individual measures. Hence, when turning to limitations of our study, our results must be interpreted on a descriptive level that warrant further, detailed studies to understand causality and underlying mechanisms and relations. Adding to that, SCC were also only assessed with one item each. This limits the precision and level of detail of the information assessed. However, we argue that the questionnaire was manageable for study participants due to that parsimony, which reduces dropout after starting the questionnaire and prevents answers on chance level due to fatigue and boredom. Anxiety and depression as the two major influential variables were not subdivided into clinical and subclinical manifestations, but assessed as continuous measure without qualitative thresholds that might distinguish clinical and subclinical populations. Hence, we do not know from the present study, whether the relation between SCC and clinical manifestation of depression and/or anxiety might be categorically different/of a different quality. As a general concern of data assessment, the study assessed data on delicate issues such as health, habits, and consumption of alcohol and nicotine. Although perceived and real anonymity is high in online surveys, we cannot rule out the risk of data distortion due to participants’ concerns about being identifiable. Also, all information is based on self-report with the additional difficulty of the anonymity of the assessment. It is, hence, impossible to verify the veracity of the data beyond simple plausibility checks. Survey data are not well suited for analyzing underlying causal mechanisms as variables of interest cannot be selectively modified with other variables being controlled for. This, together with the cross-sectional design, adds to the descriptive nature of our analyses rather than allowing for causal inferences which are impossible from the present research. As a last point, we critically note that personality, as one known influence of SCC was not assessed in the present study, but might have added additional explained variance.

## Conclusion

Our study on correlates of SCC in middle-aged adults added knowledge to the degree that middle adulthood as stage of life has been investigated as opposed to the more typical older adulthood in terms of SCC. Also, as lifestyle variables have come into focus as influencing health in general, but also cognitive performance, it seemed logical to also include lifestyle factors into these analyses (which has been done in samples with older adults already). Our findings corroborate previous research that SCC might rather be taken as an indicator of mental health, well-being and affective state than actual cognitive performance. Future research, however, should address the question of whether or not SCC are influenced differentially depending on the cognitive subdomain addressed (for example memory or selective attention). Also, whether or not *specific* foods might influence SCC (for example fish vs Mediterranean diet) warrants deeper understanding. Most of all, however, results transfer and corroborate the findings from studies with older adults to middle adulthood, that SCC predominantly reflect mental-health and psychological well-being. Their diagnostic value could mainly be exploited as an early onset of possible initial signs of change in mental health.

## Data Availability Statement

The datasets generated for this study are available on request from the corresponding author.

## Ethics Statement

Ethical review and approval was not required for the study on human participants in accordance with the local legislation and institutional requirements. Written informed consent for participation was not required for this study in accordance with the national legislation and the institutional requirements.

## Author Contributions

AM drafted the manuscript and conducted the analyses. NW, AG, and SM performed the research. All authors contributed to and have approved the final version of the manuscript.

## Conflict of Interest

The authors declare that the research was conducted in the absence of any commercial or financial relationships that could be construed as a potential conflict of interest.
